# Interesting Case of Hemicrania

**Published:** 1857-12

**Authors:** J. Levison


### INTERESTING CASE OF HEMICRANIA,

From a Carious Tooth, and Necrosed Fangs, with a highly Vascular condition of the Pulp cavity, (with a Drawing.)

BY J. L. LEVISON, D. D. S., &c.

We find an article in the October issue of the American Journal of Dental Science, with the above caption; which in some respects is rather peculiar. There are some features in the case, which it is rather difficult to understand. For instance, in the caption, "*With a highly Vascular Condition of the Pulp cavity.*" Does the writer mean that the pulp was highly vascular? Who ever saw a tooth pulp that was not vascular. But we will not impute things to him that he did not say. He says it is the "*Pulp cavity,*" that is, the chamber that contains the pulp. Now we give it up that, that is vascular too, does the "*highly vascular*" condition mean that this is one large vessel, or that this pulp cavity is divided into a great number of small ones.

About the merits of the case I do not now propose to say any thing, for I suppose if we had an intelligible description of it, it would be interesting. But the manner in which this article is gotten up is peculiar, and seems at two or three points to require a little attention.

On the first page at bottom, "but when from excitement or too great mental labor, these functions were so much deranged that constipation of the bowels was the result, then his whole *moral* being was disturbed, &c." Which was most disturbed, his *moral* or his physical being; you give us some rather strong indications of the physical disturbance, but none of moral disturbance. Did it make him swear?

It is probably just a little slip in orthography, it is spelled moral when it should have been spelled physical; such mistakes will occasionally occur. Below the middle of the second page is the following language. "We may remark, *en passant*, that this gentleman had a very large *organ of firmness*, and thus he persevered against his better sense by putting off the evil day, but in a moment of frenzy he had it extracted." Does every one who has a "*large organ of firmness*" persevere against his better sense? Is a "large organ of firmness" antagonistic to good sense? If so it would be better to drop one or the other, and which would it be best to let go, the organ of firmness, or better sense? Well, we learn from the text what this man did; when he got into a state of "*frenzy*" he *knocked* his "large organ of firmness" into a *cocked hat*, and yielded to his better sense, "and had *it* extracted." Query. Does every body have to get into a *frenzy* before they yield to their better sense?

"And had *it* extracted," had what extracted? It would be very desirable to know the whereabouts of the antecedent of "*it*." It might be the organ of firmness (probably that is what he did with it, had it extracted,) or his "better sense," or "the evil day;" any of these according to the construction of the language may do for the antecedent of "it." But whatever "it" was, "it" was extracted, and we see nothing else here that was dealt so hardly with, as that "organ of firmness,"—poor fellow.

"And we have deemed it worthy of an especial notice as a *most* well marked case of true hemicrania." The language in this sentence would be much better without the word "*most.*"

"The tooth was not decayed on the anterior surface of the crown but on one of its *lateral sides.*" What other sides could it have than "lateral sides?" Definition of lateral, is, pertaining to the side; then the word lateral in this connection is wholly superfluous. If the writer intended to tell us what side the tooth was decayed upon, he has missed his aim; but it was on one of the side sides.

On the third page near the top is the following:

"One fang exposed the body of the pulp, (the latter being inflamed and highly vascular,) and the dentine of the remaining portion of the root showed an inflamed condition." How one of the fangs could perform the feat of exposing the body of the pulp, is more than we can conceive; it is very easy to conceive how the pulp would be exposed by the removal of the fang, but according to the language, the fang must have been there, for *it* exposed the pulp. It is easy to see how the fang could hide the pulp, but not so easy to see how it would expose it. Well, we will give it up that it did it any how; but we do contend that it was a mean trick.

Then we have the very interesting announcement that the pulp was highly vascular. In the caption the *pulp cavity* was vascular. Well, this author is *some* on vascularity.

"And the dentine of the remaining portion of the root showed an inflammatory condition."

What become of the portion of root that was not remaining? Oh! that was the root that had been exposing the pulp, and it concluded to leave. Wonder if it will ever expose any thing else?

Well, really here was rather a peculiar condition of things. The tooth "acting nearly as a foreign body," (a foreign body is one which has no organic connection with the system) with a "pulp of increased vitality," and yet "nearly a foreign body." "With a fang much *absorbed* and in a state of partial *necrosis*," and the "dentine" of that part showing an *inflammatory* "condition" (necrosis and all) and the tooth "nearly a foreign body."

Here are contraries with a vengeance.

Now Mr. Smoker you will go off with a flea in *your* ear. See latter part of the article.

It is probable that it will take two articles yet from Dr. L. to explain this one, and if he goes upon the principle the Teutonic gentleman did in getting up his wrath, the third article will be *Greek.*

